# A nationwide study of Stevens–Johnson syndrome and toxic epidermal necrolysis in hospitalized pregnant women in the United States, 2009–2020

**DOI:** 10.1016/j.jdin.2024.04.002

**Published:** 2024-04-09

**Authors:** Paul Wasuwanich, Robert S. Egerman, Tony S. Wen, Kiran Motaparthi

**Affiliations:** a University of Florida College of Medicine, Gainesville, Florida; b Division of Maternal-Fetal Medicine, Department of Obstetrics & Gynecology, University of Florida College of Medicine, Gainesville, Florida; c Department of Dermatology, University of Florida College of Medicine, Gainesville, Florida

**Keywords:** autoimmune diseases, communicable diseases, epidemiology, public health, Stevens-Johnson syndrome, toxic epidermal necrosis

## Abstract

**Background:**

Stevens-Johnson syndrome (SJS) and toxic epidermal necrolysis (TEN) are rarely described in the pregnant population, and knowledge of their impact on the mother/fetus is limited.

**Objective:**

To describe SJS/TEN in pregnant women and to investigate the risk factors for developing SJS/TEN in pregnancy.

**Methods:**

We utilized hospitalization data from the 2009–2020 National Inpatient Sample. Pregnancy hospitalizations and SJS/TEN involvement were identified by ICD-9/10 codes and analyzed by chi-square and logistic regression.

**Results:**

We identified 650 pregnancies complicated by SJS/TEN requiring hospitalization. The median age was 28 years, and most were non-Hispanic White (55.2%). There were ≤10 cases associated with mortality. Most SJS/TEN cases (73.9%) occurred during the third trimester. HIV infection (OR = 9.49; *P* = .030), herpes simplex virus infection (OR = 2.49; *P* = .021), genitourinary tract infections (OR = 3.80; *P* < .001), malignant neoplasm (OR = 8.67; *P* = .031), and lupus erythematosus (OR = 41.94; *P* < .001) were associated with increased odds of developing SJS/TEN in pregnancy. Rates of preterm births were higher in the SJS/TEN cohort, 16.9% versus 8.2% (*P* < .001). Rates of pre-eclampsia, stillbirths, and post-term births were similar between the SJS/TEN versus non-SJS/TEN pregnancy cohorts.

**Limitations:**

Limited cohort size.

**Conclusions:**

SJS/TEN in pregnancy appears to be mild and is associated with favorable maternal-fetal outcomes, except for increased preterm birth.

**Capsule Summary**

• Stevens-Johnson syndrome (SJS) and toxic epidermal necrolysis (TEN) rarely occur during pregnancy and are associated with few complications and particularly rare maternal or fetal deaths.
• Genitourinary tract infections, lupus erythematosus, herpes simplex virus infection, HIV infection, and malignant neoplasms are risk factors for developing SJS/TEN in pregnancy.

## Introduction

Stevens-Johnson syndrome (SJS) and toxic epidermal necrolysis (TEN) are the most severe in the spectrum of T cell-mediated adverse drug reactions. SJS and TEN form a spectrum of the same disease and are distinguished based on involved body surface area (BSA) with BSA <10% being classified as SJS, BSA between 10% and 30% as SJS-TEN overlap syndrome, and BSA >30% as TEN.^1^ The etiology of SJS/TEN is multifactorial and involves complex interactions within the immune system.^2^ Although varying geographically, the incidence of SJS/TEN is approximately 2 cases per million people per year.^3^

The most common medications implicated in SJS/TEN include allopurinol, antiepileptic drugs, antibacterial sulfonamides, antiretroviral drugs including nevirapine, and nonsteroidal anti-inflammatory drugs.^4^^,^^5^ Certain populations are at increased risk of developing SJS/TEN including individuals with human immunodeficiency virus (HIV).^4^ The effects of pregnancy on the pathogenesis of SJS/TEN are not well understood, but SJS/TEN is thought to be rare in the pregnant population.^6^^,^^7^

In pregnant women, SJS/TEN has been described in association with preterm labor, low-birth weight, fetal respiratory distress, postpartum sepsis, vaginal stenosis, and vaginal adhesions.^8^^,^^9^ However, these associations are greatly limited by sample size and inability to compare affected patients to a population of pregnant women without SJS/TEN. We aimed to utilize the large National Inpatient Sample database to describe the demographic and clinical characteristics of SJS/TEN in pregnant women and to investigate the risk factors for developing SJS/TEN during pregnancy.

## Materials and methods

### Study population

After approval from the University of Florida Institutional Review Board (IRB), we utilized data from the National Inpatient Sample, a database that reflects inpatient stays and hospital discharges in the United States from the Healthcare Cost and Utilization Project (HCUP). A 12-year interval from January 1st, 2009 to December 31st, 2020 was selected. The year 2009 was the earliest year where SJS and TEN were codified in the National Inpatient Sample.

### Data extraction

Using International Classification of Diseases, Ninth Revision (ICD-9) and International Classification of Diseases, 10th Revision (ICD-10) codes, we identified hospitalizations involving pregnancy with and without SJS/TEN. SJS/TEN included SJS, SJS-TEN overlap syndrome, and TEN diagnoses. SJS was identified by ICD-9 diagnosis code 695.13 and ICD-10 diagnosis code L51.1; SJS-TEN overlap syndrome was identified by ICD-9 diagnosis code 695.14 and ICD-10 diagnosis code L51.3; and TEN was identified by ICD-9 diagnosis code 695.15 and ICD-10 diagnosis code L51.2.

We extracted demographic and geographic data including age, sex, race/ethnicity, household income quartile, hospital charges, region of hospital, size of hospital, and type of hospital (rural, urban nonteaching, or urban teaching). The following clinical data were obtained: length of hospital stay, all-cause mortality, trimester, diabetes mellitus type I/II, obesity, hepatitis B, hepatitis C, HIV infection, *Mycoplasma* infection, herpes simplex virus infection, genitourinary tract infection, malignant neoplasm, cutaneous autoimmune diseases, history of injection drug use, pre-eclampsia, and fetal heart rate/rhythm abnormality. We also collected birth data including live births, stillbirths, preterm births, and post-term births. Hospital charges were reported in United States dollars. Cutaneous autoimmune diseases include lupus erythematosus, dermatomyositis, pemphigoid, and pemphigus. History of injection drug use was determined indirectly; hospitalizations with the diagnosis of dependence on drugs commonly injected intravenously including amphetamines, opioids, sedatives, hallucinogens, or combinations of these were considered to reflect a history of injection drug use.

### Statistical analysis

Normality of quantitative data was tested using the Kolmogorov–Smirnov test. The non-normal data were summarized using the median and interquartile range (IQR) and analyzed with the Mann–Whitney U test. Normally distributed data were summarized with means and standard deviation and analyzed with the Student’s t-test. Frequencies were compared using the chi-squared test. Risk factors for developing SJS/TEN in pregnancy were analyzed by univariable (crude) logistic regression and multivariable (adjusted) logistic regression models. The dichotomous outcomes were SJS/TEN versus no SJS/TEN during hospitalization. Results of logistic regression were reported by odds ratio (OR) with 95% confidence intervals (CIs). ORs were derived from exponentiated beta logistic regression coefficients from the logistic regression. Additionally, as some studies have reported that the accuracy of SJS/TEN diagnoses are potentially unreliable for hospitalizations with short lengths of stay,^10^ we created 2 cohorts for analysis, one utilizing the original dataset and the other excluding all cases with hospitalizations less than 3 days in length. We performed the same statistical analyses on both cohorts and reported the results of the former cohort in the main text and the latter cohort in the Supplementary Material, available via Mendeley at https://data.mendeley.com/datasets/rcb6mbr893/1.

Using discharge weights provided by HCUP to present the true number of hospitalizations in the United States, all results reported were weighted. Results for a category that contain greater than 0 but fewer than 10 hospitalizations were displayed as ≤10 due to the data use privacy policy of HCUP. *P*-values were calculated from weighted data using actual numbers regardless of whether ≤10 hospitalizations were reported. All reported *P*-values were two-tailed *P*-values. Statistical significance was defined as *P* < .05. Statistical analyses were performed using R program (R Core Team (2020). R: A language and environment for statistical computing. R Foundation for Statistical Computing. URL https://www.R-project.org/.).

## Results

Out of a total of 49,139,435 pregnancy-related hospitalizations in the United States between 2009 and 2020, we identified 650 hospitalizations for SJS/TEN during pregnancy and 49,138,785 hospitalizations during pregnancy without SJS/TEN. In the SJS/TEN cohort, the median age was 28 years with an IQR of 23-32 years. The race/ethnic distribution was 359 (55.2%) non-Hispanic White, 118 (18.2%) non-Hispanic Black, 84 (12.9%) Hispanic, 25 (3.8%) Asian or Pacific Islander, ≤10 Native American, and 59 (9.1%) other/unknown. The majority of hospitalizations for SJS/TEN occurred in the Southern region of the United States (45.7%), and 68.6% of hospitalizations occurred in urban teaching hospitals. Between cohorts of pregnant women with SJS/TEN and pregnant women without SJS/TEN, there were no statistically significant differences in age, race/ethnicity, regional distribution, hospital type, or hospital bedsize (*P* > .05). However, the median cost of hospitalization was significantly higher in the cohort of pregnant women with SJS/TEN compared to the cohort of pregnant women without SJS/TEN at $16,605 (IQR: $9869-$29,483) versus $13,761 (IQR: $8922-$21,495), respectively (*P* = .006). These results are summarized in Table I. Results that reflect the exclusion of hospitalizations less than 3 days in length are summarized in Supplementary Table I, available via Mendeley at https://data.mendeley.com/datasets/rcb6mbr893/1.

**Table I:** Demographic, geographic, and social characteristics of pregnancies with and without Stevens-Johnson syndrome (SJS) and toxic epidermal necrolysis (TEN). National Inpatient Sample, Healthcare Cost, and Utilization Project (HCUP), 2009-2020

Characteristics	SJS/TEN pregnancy	Non-SJS/TEN pregnancy	*P*-value
Number of hospitalizations, *N*	650	49,138,785	
Age, year, median (IQR)	28 (23-32)	28 (24-32)	.848
Race/Ethnicity			.264
Non-Hispanic White, *N* (%)	359 (55.2)	23,793,160 (48.4)	
Non-Hispanic Black, *N* (%)	118 (18.2)	7,162,349 (14.6)	
Hispanic, *N* (%)	84 (12.9)	9,791,950 (19.9)	
Asian or Pacific Islander, *N* (%)	25 (3.8)	2,532,955 (5.2)	
Native American, *N* (%)	≤10	368,133 (0.7)	
Other/unknown, *N* (%)	59 (9.1)	5,490,238 (11.2)	
Region of hospital			.237
Northeast, *N* (%)	115 (17.7)	7,868,651 (16.0)	
Midwest, *N* (%)	123 (18.9)	10,290,340 (20.9)	
South, *N* (%)	297 (45.7)	19,151,271 (39.0)	
West, *N* (%)	115 (17.7)	11,828,524 (24.1)	
Type of hospital			.057
Rural, *N* (%)	74 (11.4)	4,817,580 (9.8)	
Urban non-teaching, *N* (%)	130 (20.0)	14,431,013 (29.4)	
Urban teaching, *N* (%)	446 (68.6)	29,671,680 (60.4)	
Hospital bedsize			.146
Small, *N* (%)	64 (9.8)	7346051 (14.9)	
Medium, *N* (%)	172 (26.5)	14139675 (28.8)	
Large, *N* (%)	413 (63.5)	27434547 (55.8)	

*IQR*, Interquartile range.

We examined the clinical characteristics of pregnancies with and without SJS/TEN. Among the 650 pregnancies with SJS/TEN, there were ≤10 cases of maternal mortality, and the median length of hospital stay was found to be 3 days with an IQR of 2-5 days. Among the hospitalizations with available trimester data, the distribution was as follows: 8.7% first trimester, 17.4% second trimester, and 73.9% third trimester. Compared to the pregnant cohort without SJS/TEN, the pregnant cohort with SJS/TEN had significantly greater frequencies of HIV infection [≤10 versus 32,454 (0.1%); *P* = .002], *Mycoplasma* infection [≤10 versus 2456 (<0.1%); *P* < .001], herpes simplex virus infection [35 (5.4%) versus 1,055,407 (2.1%); *P* = .011], genitourinary tract infection [64 (9.8%) versus 1,106,462 (2.3%); *P* < .001], malignant neoplasm [≤10 versus 36,030 (0.1%); *P* = .003], cutaneous autoimmune disease [54 (8.3%) versus 85,685 (0.2%); *P* < .001], and lupus erythematosus [54 (8.3%) versus 82,917 (0.2%); *P* < .001]. However, compared to the pregnant cohort without SJS/TEN, the pregnant cohort with SJS/TEN had significantly lower frequencies of fetal heart rate/rhythm abnormalities [49 (7.5%) versus 7,144,329 (14.5%); *P* = .024]. There were no statistically significant differences in the frequencies of diabetes, obesity, hepatitis B or C, intravenous drug use, or pre-eclampsia between pregnant women with SJS/TEN and pregnant women without SJS/TEN (*P* > .05) (Table II).

**Table II:** Clinical characteristics of pregnancies with and without Stevens-Johnson syndrome (SJS) and toxic epidermal necrolysis (TEN). National Inpatient Sample, Healthcare Cost and Utilization Project (HCUP), 2009-2020

Characteristics	SJS/TEN pregnancy	Non-SJS/TEN pregnancy	*P*-value
Number of hospitalizations, *N*	650	49,138,785	
Length of stay, days, median (IQR)	3 (2-5)	2 (2-3)	**<.001**
Mortality, *N* (%)	≤10	8190 (0.2)	**<.001**
Metabolic disorders			
Diabetes, *N* (%)	20 (3.1)	634,837 (1.3)	.077
Obesity, *N* (%)	40 (6.2)	2,971,577 (6.0)	.980
Infections			
Hepatitis B or C, *N* (%)	≤10	277,686 (0.6)	.139
HIV positive, *N* (%)	≤10	32,454 (0.1)	**.002**
Mycoplasma infection, *N* (%)	≤10	2456 (<0.1)	**<.001**
Herpes simplex virus infection, *N* (%)	35 (5.4)	1,055,407 (2.1)	**.011**
Genitourinary tract infection, *N* (%)	64 (9.8)	1,106,462 (2.3)	**<.001**
Other			
Malignant neoplasm, *N* (%)	≤10	36,030 (0.1)	**.003**
Cutaneous autoimmune disease, *N* (%)	54 (8.3)	85,685 (0.2)	**<.001**
Lupus, *N* (%)	54 (8.3)	82,917 (0.2)	**<.001**
Intravenous drug use, *N* (%)	15 (2.3)	453,403 (0.9)	.098
Pregnancy complications			
Pre-eclampsia, *N* (%)	45 (6.9)	2,601,189 (5.3)	.403
Fetal heart rate/rhythm abnormality, *N* (%)	49 (7.5)	7,144,329 (14.5)	**.024**
Number of deliveries, *N*	378	44,355,925	
Length of stay, days, median (IQR)	2 (2-3)	2 (2-3)	**.048**
Live birth, *N* (%)	373 (98.7)	44,027,841	.555
Still birth, *N* (%)	≤10	345,179 (0.8)	.591
Preterm birth, *N* (%)	64 (16.9)	3,636,024 (8.2)	**.005**
Post-term birth, *N* (%)	35 (9.3)	5,583,620 (12.6)	.372

Statistically significant *P*-values (less than .05) are bolded.

*HIV*, Human immunodeficiency virus; *IQR*, interquartile range.

Among the 650 pregnant women who had SJS/TEN, there were 378 (58.2%) deliveries. There were ≤10 cases of stillbirths, a rate which was not significantly higher than the stillbirth rate of the non-SJS/TEN deliveries (*P* = .555). Preterm births were more common in the pregnant cohort with SJS/TEN compared to the pregnant cohort without SJS/TEN [64 (16.9%) versus 3,636,024 (8.2%); *P* = .005]. There were also no significant differences in the rates of live births and post-term births between the 2 cohorts (*P* > .05). Median length of stay for pregnant women with SJS/TEN who were delivering was 2 days with an IQR of 2–3 days (Table II). Results that reflect the exclusion of hospitalizations less than 3 days in length are summarized in Supplementary Table II, available via Mendeley at https://data.mendeley.com/datasets/rcb6mbr893/1.

We investigated risk factors associated with the development of SJS/TEN during pregnancy using a multivariable (adjusted) logistic regression model. HIV infection (OR = 9.49; 95% CI = 1.24-7.24; *P* = .030), herpes simplex virus infection (OR = 2.49; 95% CI = 1.15-5.41; *P* = .021), genitourinary tract infection (OR = 3.80; 95% CI = 2.01-7.16; *P* < .001), malignant neoplasm (OR = 8.67; 95% CI = 1.22-61.39; *P* = .031), and lupus erythematosus (OR = 41.94; 95% CI = 20.78-84.66; *P* < .001) were significantly associated with increased odds of developing SJS/TEN in pregnant women. Age, race/ethnicity, obesity, diabetes, hepatitis B or C, intravenous drug use, and pre-eclampsia were not significantly associated with developing SJS/TEN (*P* > .05) (Table III). Results that reflect the exclusion of hospitalizations less than 3 days in length are summarized in Supplementary Table III, available via Mendeley at https://data.mendeley.com/datasets/rcb6mbr893/1.

**Table III:** Risk factors for the development of Stevens-Johnson syndrome (SJS) and toxic epidermal necrolysis (TEN) in pregnant women. National Inpatient Sample, Healthcare Cost and Utilization Project (HCUP), (2009-2020)

Risk Factors		Crude odds ratio (95% CI)	Crude *P*-value	Adjusted odds ratio (95% CI)	Adjusted *P*-value
Demographics					
Age	≥30 (vs < 30)	0.99 (0.70-1.41)	.973	0.96 (0.65-1.28)	.821
Race/Ethnicity	White (vs other)	1.31 (0.92-1.88)	.137	1.40 (0.97-2.01)	.071
Metabolic disorders					
Diabetes	Yes (vs no)	2.39 (0.88-6.47)	.086	2.25 (0.82-6.16)	.114
Obesity	Yes (vs no)	1.01 (4.94-2.06)	.980	0.81 (0.37-1.78)	.597
Infections					
Hepatitis B or C	Yes (vs no)	2.75 (0.68-11.11)	.156	1.84 (0.41-8.24)	.423
HIV Positive	Yes (vs no)	1.17 (1.64-8.40)	**.014**	9.49 (1.24-7.24)	**.030**
Herpes simplex virus infection	Yes (vs no)	2.60 (1.21-5.57)	**.014**	2.49 (1.15-5.41)	**.021**
Genitourinary tract infection	Yes (vs no)	4.75 (2.68-8.43)	**<.001**	3.80 (2.01-7.16)	**<.001**
Other					
Malignant neoplasm	Yes (vs no)	10.57 (1.48-75.62)	**.019**	8.67 (1.22-61.39)	**.031**
Lupus	Yes (vs no)	53.85 (2.90-99.99)	**<.001**	41.94 (20.78-84.66)	**<.001**
Intravenous drug use	Yes (vs no)	2.54 (0.81-7.97)	.111	1.65 (0.48-5.69)	.429
Pregnancy complications					
Pre-eclampsia	Yes (vs no)	1.33 (0.68-2.63)	.405	1.27 (0.63-2.55)	.502

Adjusted logistic regression model adjusted for all variables.

Statistically significant *P*-values (less than .05) are bolded.

*CI*, Confidence interval; *HIV*, human immunodeficiency virus.

## Discussion

In this study, we describe the largest cohort of pregnant women with SJS/TEN to date, significantly expanding upon an earlier preliminary study from our group.^11^ The results of this present study reaffirm the significant findings of the earlier study but with some notable differences. To address potential concerns regarding the reliability of SJS/TEN diagnoses in the National Inpatient Sample,^10^^,^^11^ we reported data from the original National Inpatient Sample as well as more selective data that excluded hospitalizations less than 3 days in length.

In studies of SJS/TEN in general, the overall mortality rate has been reported near 5.4% for SJS and 15.3% for TEN.^12^ Applying the more conservative mortality rate of 5.4% to the cohort of 650 pregnant women, we would have expected approximately 35 deaths compared to ≤10 identified. Even in the selective cohort of 346 pregnant women which excluded hospitalizations with length of stays less than 3 days, we would have expected approximately 19 deaths compared to ≤10 in this cohort. The low rate of mortality in this cohort of pregnant women with SJS/TEN is consistent with prior descriptions of pregnant women with SJS/TEN.^6^^,^^13^^,^^14^ Pregnant women who develop SJS/TEN may experience a milder form of the disease, possibly due their relatively immunosuppressed state. Additionally, their relatively young age may also contribute to a higher survival rate when compared to the general SJS/TEN population. Furthermore, the risk of developing SJS/TEN appears to be greatest in the third trimester of pregnancy and lowest in the first trimester. Pregnant women in the third trimester typically receive close follow up for their prenatal care, approximately every 1–3 weeks, potentially allowing for earlier care for SJS/TEN and lower mortality.

Similar to our preliminary study,^11^ we found that the presence of genitourinary tract infection, malignant neoplasm, and cutaneous autoimmune disease to be significantly associated with increased odds of developing SJS/TEN in pregnancy. Genitourinary tract infections are common in pregnant women and are generally treated with antibiotics which have been frequently implicated as causative agents in SJS/TEN.^15^^,^^16^ Moreover, nonpregnant patients with malignant neoplasms are known to have greater risk of developing SJS/TEN, potentially due to increased exposure to culprit medications.^17^ In this study, we explored the relationship between cutaneous autoimmune disease and the development of SJS/TEN in pregnancy. Upon further investigation, all cutaneous autoimmune disease in the SJS/TEN pregnant group was due to lupus erythematosus. The association of lupus erythematosus and SJS/TEN is unclear in the nonpregnant population and has never been studied in the pregnant population.^18^^,^^19^ As acute cutaneous manifestations of lupus erythematosus and SJS/TEN may demonstrate similar clinical features, the potential for misdiagnosis exists,^18^ but physicians should have an increased index of suspicion for SJS/TEN in a pregnant woman with a past medical history of lupus erythematosus.

In addition, HIV infection and herpes simplex virus infection were significantly associated with SJS/TEN in pregnant women, in contrast to our preliminary study which was limited by sample size.^11^ A systematic review by Sharma found HIV to be common in pregnant women with SJS/TEN, and nevirapine was the most common offending drug among patients receiving antiretroviral therapy.^8^ Patients with HIV infections are more likely to develop drug eruptions, potentially due to the loss of skin-protective regulatory CD4+CD25+ T cells.^20^ The relationship between herpes simplex virus infection and SJS/TEN in pregnancy has not been previously studied.^21^, ^22^, ^23^

In contrast to the preliminary study, we did not find diabetes to be significantly associated with the development of SJS/TEN in pregnancy. In the general SJS/TEN population, diabetes was identified as an independent risk factor for SJS/TEN.^24^ As our preliminary study focused on the most recent years of the National Inpatient Sample database, the difference in this result may be explained by the increase in prepregnancy diabetes by 27% from 2016 to 2021.^25^

Stillbirths have been described in pregnant women with SJS/TEN.^6^ Among the 378 pregnant women with SJS/TEN who delivered within the study period, there were ≤10 cases of stillbirths, similar to the cohort of pregnant women without SJS/TEN who delivered. The rate of stillbirth in the United States, as reported by the Centers for Disease Control and Prevention (CDC), is approximately 0.057%,^26^ comparable to the rate of stillbirth in the control group of pregnant women without SJS/TEN. Therefore, SJS/TEN is not associated with an increased risk of stillbirth. However, the frequency of preterm births among pregnant women with SJS/TEN is significantly higher by more than two-fold compared to the control group. Premature/preterm labor has been sporadically described in pregnant women with SJS/TEN but has never been examined in comparison to pregnant patients without SJS/TEN.^8^

### Limitations

Due to the nature of the National Inpatient Sample database, standardization of diagnostic criteria is lacking. We rely on the professional judgement of physicians in entering the most appropriate diagnostic codes. Additionally, the potential for miscoding exists; we assumed that any miscoded diagnoses would be distributed randomly between the cohorts, without statistical impact. Due to privacy requirements by HCUP, we were not able to report exact numbers for any category that contained 10 or fewer hospitalizations. Furthermore, the National Inpatient Sample database does not provide data on medication history or medications used during the hospitalization, thus, we were unable to directly investigate the causal drugs for SJS/TEN in this cohort. Statistically significant results that involved 10 or fewer hospitalizations were not emphasized due to the possibility of error in the statistical model when analyzing small sample sizes.

## Conclusion

SJS/TEN is rare in the pregnant population and is associated with generally good outcomes. Rates of preterm birth were notably higher in this population, but rates of mortality in the mother and infant were very low. Most SJS/TEN cases occurred in the third trimester. Risk factors including genitourinary tract infections, lupus erythematosus, herpes simplex virus infection, HIV infection, and malignant neoplasms were associated with an increased odds of SJS/TEN in pregnancy. Vigilance for pregnant patients with these risk factors is warranted.

## Conflicts of interest

None disclosed.
